# A Comprehensive Review of the Covalent Immobilization of Biomolecules onto Electrospun Nanofibers

**DOI:** 10.3390/nano10112142

**Published:** 2020-10-27

**Authors:** Soshana Smith, Katarina Goodge, Michael Delaney, Ariel Struzyk, Nicole Tansey, Margaret Frey

**Affiliations:** 1 Department of Fiber Science and Apparel Design, Cornell University, Ithaca, NY 14853, USA; keg222@cornell.edu (K.G.); nlt36@cornell.edu (N.T.); margaret.frey@cornell.edu (M.F.); 2 Robert Frederick Smith School of Chemical & Biomolecular Engineering, Cornell University, Ithaca, NY 14853, USA; md839@cornell.edu (M.D.); aas324@cornell.edu (A.S.)

**Keywords:** nanofiber, biomolecule, enzyme, covalent immobilization, crosslinker, electrospinning

## Abstract

Biomolecule immobilization has attracted the attention of various fields such as fine chemistry and biomedicine for their use in several applications such as wastewater, immunosensors, biofuels, et cetera. The performance of immobilized biomolecules depends on the substrate and the immobilization method utilized. Electrospun nanofibers act as an excellent substrate for immobilization due to their large surface area to volume ratio and interconnectivity. While biomolecules can be immobilized using adsorption and encapsulation, covalent immobilization offers a way to permanently fix the material to the fiber surface resulting in high efficiency, good specificity, and excellent stability. This review aims to highlight the various covalent immobilization techniques being utilized and their benefits and drawbacks. These methods typically fall into two categories: (1) direct immobilization and (2) use of crosslinkers. Direct immobilization techniques are usually simple and utilize the strong electrophilic functional groups on the nanofiber. While crosslinkers are used as an intermediary between the nanofiber substrate and the biomolecule, with some crosslinkers being present in the final product and others simply facilitating the reactions. We aim to provide an explanation of each immobilization technique, biomolecules commonly paired with said technique and the benefit of immobilization over the free biomolecule.

## 1. Introduction

Nanofibers are favored as a substrate for biomolecule immobilization because of their high surface to volume ratio, low barrier to diffusion and interconnected pore network [1,2]. These factors allow researchers to have a much higher biomolecule loading compared to other polymer substrates such as beads and films. Nanofibers have been used to immobilize a variety of biomolecules such as enzymes, DNA, aptamers and proteins. The porous nature of the membrane allows for easy diffusion to the nanofiber surface leading to greater biomolecule retention on the large available surface area [3,4]. Researchers have found enzyme activity retention was inversely proportional to fiber diameter and attributed this enhanced activity retention to reduced interactions between molecules on the surface of the material and diminished boundary layers for diffusion [5,6,7,8]. In addition to improved activity retention, researchers have also noted enhanced pH, temperature and storage stability [9], as well as increased cell capture, growth and proliferation [10] when utilizing nanofibers for biomolecule retention.

**Figure 1:**
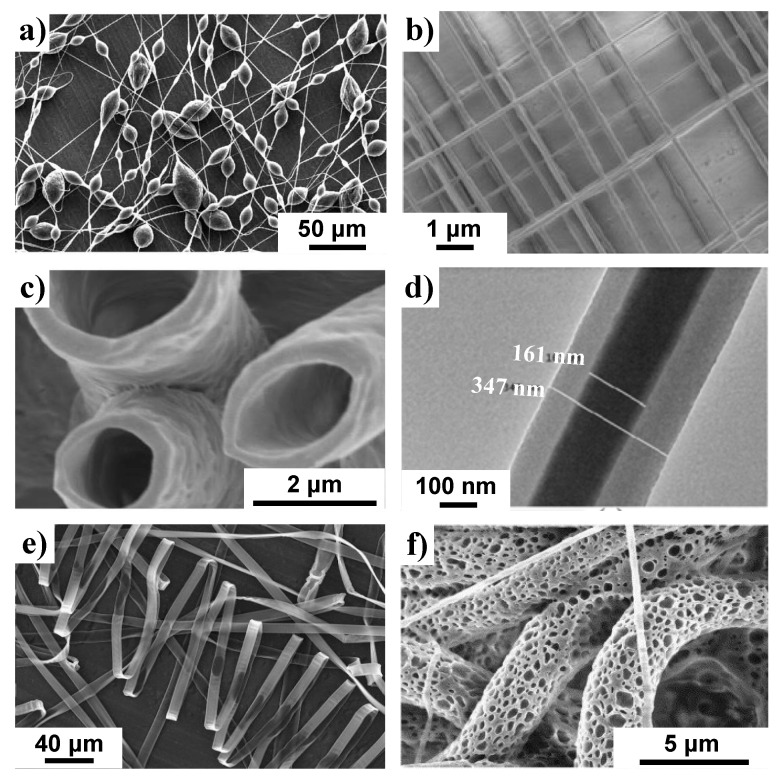
Different fiber morphologies achieved by various researchers using the electrospinning technique: (**a**) beaded; (**b**) aligned; (**c**) hollow; (**d**) core-sheath; (**e**) flat ribbon; (**f**) porous. Reproduced with permission from [13], Copyright John Wiley & Sons, Inc., 2007. Reproduced from [14], Copyright Elsevier, 2012. Reproduced from [15], Copyright John Wiley & Sons, Inc., 2007. Reproduced from [16], Copyright Springer Nature, 2011. Reproduced from [17,18], Copyright Elsevier, 2008, 2007.

There are many ways to manufacture the needed nanofibers such as self-assembly, phase separation and electrospinning [11]. Electrospinning presents a facile, inexpensive way to make highly tunable nanofibers allowing researchers great control over mat thickness and composition, surface area to volume ratio, and intra-/inter- pore size distribution. The usual electrospinning set-up consists of three main parts: a syringe filled with polymer solution, a grounded collector, and a voltage source providing electrical charge to the syringe needle. Using a pump, the spinning solution is gradually forced from the syringe into the needle. The application of the electric charge on the needle creates an electric field gradient between the needle and grounded collector. This difference creates a pendant-like droplet of the spinning fluid called a Taylor cone, which once ejected begins to whip and elongate until it reaches the collector plate. The resultant fibers have a large surface area with the typical specific surface area being 10 m^2^/g for fiber diameters around 500 nm and 1000 m^2^/g for diameters around 50 nm [12]. The final fibers can have a variety of morphologies including beaded, aligned, hollow, core-shell, flat-ribbon and porous (Figure 1a–f). The final fiber morphology and properties are impacted by various polymer parameters such as type of polymer used, polymer molecular weight, surface tension, conductivity and volatility of the spinning solution. Operation conditions such as applied voltage, feed rate, spinneret diameter and distance between the spinneret and the collecting substrate are also significant. Finally, one must consider environmental factors such as air temperature, humidity and air speed. Mastering a well-balanced application of all these factors has allowed researchers to use electrospinning as an easy method for creating substrates for biomolecule immobilization.

Immobilization of biomolecules onto nanofibers can be done in a variety of ways: encapsulation [19,20,21,22], adsorption [23,24,25,26,27,28] and covalent bonding. Encapsulated biomolecules are not attached to the nanofiber surface but instead entrapped in the polymer network. The desired biomolecule is added to the spinning solution and becomes immobilized in the polymer matrix during the electrospinning process. This method works well for immobilizing biocatalysts, such as enzymes, as well as encapsulating drugs and vitamins. This method improves enzyme stability as well as reduces leaching into the surrounding solution or denaturation of the enzyme. However, because the biomolecule is encapsulated in the nanofiber, high mass transfer resistance leads to large amounts of the biomolecule not being utilized fully [29]. Biomolecule immobilization by adsorption is one of the most straightforward methods of immobilization. The biomolecule and the nanofiber substrate are placed in solution for a fixed amount of time and then rinsed with buffer solution to remove any unadsorbed biomolecules. The mechanism for immobilization is based on weak interactions such as Van der Waals forces, hydrophobic and electrostatic interaction. This simple process has many advantages: it is reagent free, non-destructive to both the substrate and biomolecule, low cost and easily reversible. However, because the biomolecules are loosely bound to the polymer matrix, they can be easily desorbed from the nanofiber surface due to changes in temperature, pH and surface charge [30].

A major drawback to the two previously mentioned strategies is the ability of the biomolecule to leach into the surrounding aqueous environment [9]. In order to combat this shortcoming, researchers can also covalently bind the biomolecule to the nanofiber surface [31]. During immobilization, stable complexes are formed between the functional groups of the substrate and the functional groups of the biomolecule. The functional groups that can partake in the reaction are the amino, carboxylic, thiol, imidazole, indole and hydroxyl groups [32]. The type of immobilization reaction used will be determined by which functional group is available. The binding procedure of the biomolecule onto the nanofiber substrate can go through two steps: (1) direct reaction onto the substrate (Section 2, Section 3 and Section 8) or (2) activation of the surface through the use of crosslinkers (Section 4, Section 5, Section 6 and Section 7).

**Figure 2:**
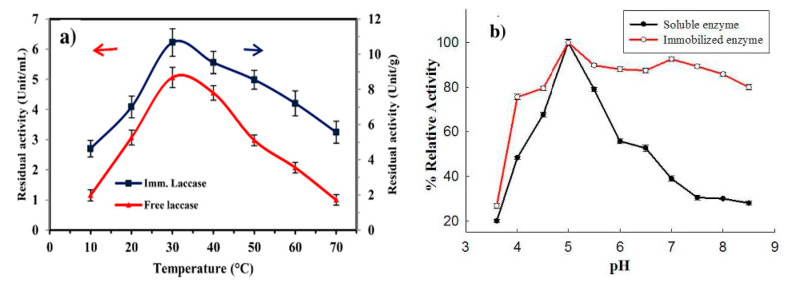
(**a**) Effect of temperature on the activity of free and immobilized laccase; (**b**) Effect of pH on the stability of free and immobilized β-amylase. Reproduced with permission from [35], Copyright American Chemical Society, 2017. Reproduced from [36], Copyright Elsevier, 2017.

Because the biomolecules are covalently bonded to the fiber surface, there are no mass transfer limitations of substrate to biomolecule active site as seen with encapsulation or leaching as seen with adsorption. Covalent immobilization of enzymes typically leads to increased stability of enzymes; allowing the enzymes to be more stable over a wider array of temperatures and pH values compared to free enzymes (Figure 2a,b). However, a major drawback of covalent bonding is the significant loss of enzymatic activity that can occur after immobilization. Depending on the technique used, enzymes can lose up to 98% of their enzymatic activity post-immobilization. This loss in activity is attributed to changes to the active sites of the enzyme or immobilization of the enzyme in a particular orientation which can make active sites unavailable [33]. Researchers can quantify this loss by noting the change in specific activity, activity of an enzyme per milligram of total protein, after immobilization. Researchers will also frequently discuss relative/residual activity which is the ratio of the initial activity of the enzyme over the activity of the immobilized enzyme [34].

**Figure 3:**
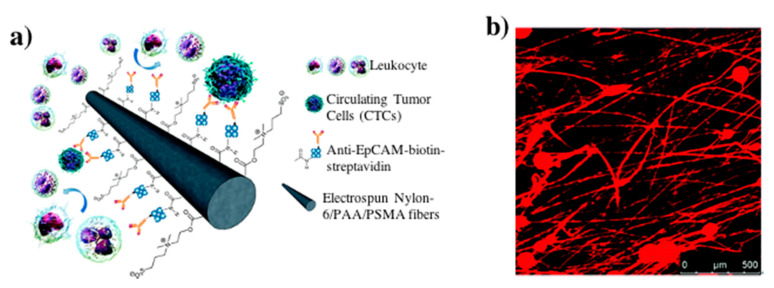
(**a**) Schematic representation of CTC capture of electrospun triple-blend fibrous mats from blood specimens, with repulsive ability against leukocyte adsorption; (**b**) Confocal images of Fluorescein isothiocyanate (FITC)-streptavidin-modified after the immunosorbent reaction with Cy5-biotin. Reproduced with permission from [41]. Copyright Royal Society of Chemistry, 2016.

Covalent immobilization of other biomolecules, including DNA and aptamers, is commonly utilized in immunosensors for specific antigen capture (Figure 3a). Covalent immobilization allows for vertical orientation of the DNA probe, thus facilitating greater interaction with the desired antigen. This leads to greater antigen capture and attachment. Unlike with enzymes this attachment is not quantified. Researchers instead will sometimes use florescent markers to confirm uniform coating of the desired biomolecule or capture antigen onto the nanofiber (Figure 3b) [37,38,39,40].

The present review aims to summarize the various methods that can be utilized to immobilize biomolecules onto electrospun nanofibers. These methods include direct covalent immobilization, the use of crosslinkers and newly emerging “click” chemistry. We seek to highlight and compare each method, noting their benefits and drawbacks for specific types of biomolecules. We have also provided a table (Table 1) at the end of this review categorizing the use of each polymer type with the immobilization technique for easy reference. 

## 2. Direct Immobilization

**Figure 4:**
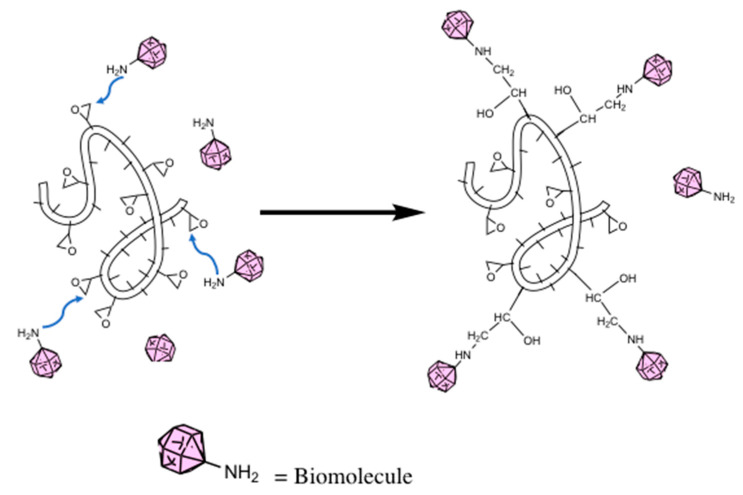
Schematic representation of epoxide ring opening mechanism for the covalent attachment of enzymes onto nanofibers.

Certain polymers can covalently bind with biomolecules without the need for surface modification or crosslinkers. These polymers are typically strong electron acceptors which can undergo rapid reaction at physiological pH values. For biomolecule attachment, copolymers containing cyclic structures such as poly(glycidyl methacrylate-co-methylacrylate (P(GMA-co-MA)) and poly(styrene-co-maleic anhydride) (PSMA) are typically used. PGMA contains highly strained epoxy ring groups along the polymer backbone; by their nature, epoxides tend to be more electrophilic due to this strained ringed system. The amine groups present on the biomolecule attack the electrophilic carbon next to the epoxide oxygen, resulting in a negative charge on the oxygen and a positive charge on the nitrogen. The oxygen’s extra pair of electrons removes a hydrogen from the ammonium nitrogen, resulting an alcohol group and an amide group (Figure 4) [42,43,44]. Liu et al. [45,46] used electrospun PGMA fibers in both of their works to immobilize lipase, combining PGMA with other materials to improve enzyme activity and stability. Liu et al. found using feather polypeptide (FP) instead of PEO resulted in a lower K_m_ value (0.19 vs. 0.218 g/mL) indicating a greater affinity between enzyme and substrate. The Michaelis–Menten constant, K_m_, represents the inverse relationship to substrate affinity [47]. A high K_m_ value indicates an enzyme’s low affinity to a particular substrate. Both works showed the immobilized lipase demonstrated better temperature and pH stability compared to the free enzyme. In both cases, the lipase was able to retain greater enzyme activity over a wide range of conditions. This is attributed to the multipoint connection between lipase and nanofiber mat leading the conformation of lipase being stabilized and the extensional deformation of the peptide is reduced. Comparing the two works, the FP-containing mat showed a greater pH tolerance with an optimal pH of 6.0 compared to 7.0 for the PEO-containing membrane. Similarly, at elevated temperatures the FP-containing membrane showed greater tolerance with 65% relative activity compared to 45% for the PEO-containing membrane and 25% for the free enzyme. In addition, the FP-containing functionalized mat showed higher reusability and organic solvent stability due to the biocompatibility of FP. 

Epoxide containing groups can also be grafted onto the base polymer if it lacks the necessary group. Oktay et al. grafted PGMA brushes onto PVA nanofibers using atom transfer radical polymerization (ATRP), which facilitated immobilization of α-amylase on the fiber surface [48]. Researchers noted nanofibers containing epoxy-activated functional groups are almost-ideal supports for immobilization leading to great storage stability compared to other techniques. Free amylase lost all of its activity within 15 days of storage in phosphate buffer (0.02 M pH 6.9) at 4 °C according to Türünç et al. [49]. However, immobilized α-amylase onto PGMA brushes lost only 23% activity. The majority of the loss (90%) occurred within the first 5 days; with minimal activity loss thereafter. Alternatively, researchers have added epoxide groups by grafting epichlorohydrin (3-chloro-1,2-epoxypropane) into their polymers via an available hydroxyl group [50,51,52]. The highly reactive halogen end of the epichlorohydrin molecule will couple with the reactive end of the hydroxyl group on the nanofiber under alkaline conditions, leaving an epoxide tether for biomolecule immobilization [53]. PAN/poly-(6-O-vinylsebacoyl d-glucose) [poly-OVSEG] nanofibers were used to immobilize catalase using epichlorohydrin; noting a similar activity retention to Oktay et al. after 30 days [51]. Similar to the previous paragraph, researchers using epoxide grafted mats noted an increase in temperature stability of the immobilized enzyme compared to the free enzyme especially at higher temperatures. For example, Türünç et al. noted a 10% increase in relative enzyme activity compared to free α-amylase at 60 °C. Comparably, Li et al. found a 30% increase in relative activity compared to free catalase at 60 °C.

**Figure 5:**
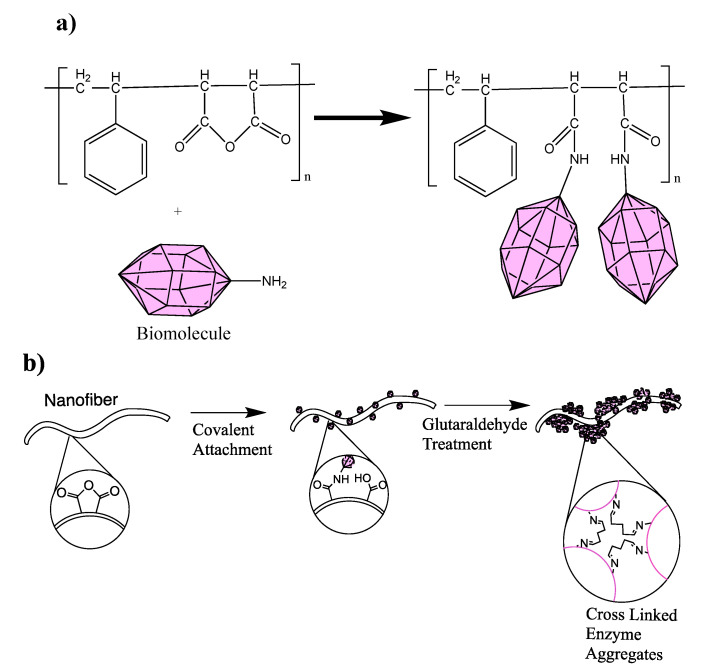
(**a**) Schematic of covalent attachment of biomolecules onto maleic anhydride functional group; (**b**) Covalent attachment of enzymes and enzyme aggregate coating. Adapted from [56].

PSMA contains a maleic anhydride group which facilitates the bonding of biomolecules to the nanofiber. The nucleophilic amine groups of the biomolecule attack the carbon bond of the maleic anhydride displacing the pi-bond temporarily resulting in a tetrahedral intermediate. The pi-bond is then reformed resulting in the elimination of the carboxylate group and reaction to the amine group to attach two biomolecules per maleic anhydride (Figure 5a). PSMA nanofibers are useful for immobilizing enzymes such as lipase, trypsin, and carbonic anhydrase among others [54]. These reactions typically take place in a sodium phosphate buffer solution with pH between 6.8 and 7.8 for approximately one hour. Because PS-PSMA nanofibers are hydrophobic, they do not disperse well in aqueous solution. To aid in dispersion, researchers rinse the nanofiber mats in alcohol. Improved dispersion leads to higher enzyme loading compared to non-alcohol treated nanofiber mats. Nair et al. achieved 42.4 μg/g nanofiber of immobilized lipase onto alcohol treated PSMA nanofibers compared to 5.6 μg/g nanofiber for the as-spun fibers. After immobilization, researchers noted an 83% loss in enzyme activity, which is far lower than activity loss seen compared to other techniques that will be discussed later in this paper [55]. This reduction in activity associated with epoxide opening covalent bonding is typically attributed to steric hindrance which limits nanofiber-enzyme interactions. Because of the hydrophobic nature of the PSMA nanofiber and increased steric hindrance, researchers have found unmodified PSMA nanofibers can have activity retention as low as 2% [54]. One way researchers have sought to resolve this issue is through the use of enzyme aggregates. In this process first utilized by Kim et al., enzymes are covalently bonded to the polymer nanofiber substrate as previously mentioned, followed by crosslinking of additional molecules onto the seed enzyme using glutaraldehyde treatment (Figure 5b) [56]. Enzyme aggregation increases enzyme loading which will then increase overall enzyme activity for the fiber mat though the activity retention for each individual enzyme remains low. For example, Jun et al. [57] immobilized carbonic anhydrase onto PSMA nanofibers using both monolayer covalent conjugation (CA) and also enzyme aggregation (EPC). Both techniques resulted in a 90% reduction in specific activity; 0.100 and 0.098 units per mg enzyme respectively compared to 1.103 units per mg enzyme for the free enzyme. Though the CA and EPC immobilized enzymes had the same specific activity, the overall initial activity of the EPC fibers was 79 times (1.14 × 10^–3^ and 90.0 × 10^–3^ units per mg nanofibers) higher due to an increase in enzyme loading (11.4 and 916 μg enzyme per mg nanofibers). These enzyme aggregate fibers typically result in excellent storage stability, retaining over 60% initial activity retention after hundreds of days of rigorous shaking. Though this technique is effective in high enzyme loading and activity, it has the potential to be extremely expensive due to the large amounts of biomaterial that need to be used, making this technique less than ideal for large-scale application. 

**Figure 6:**
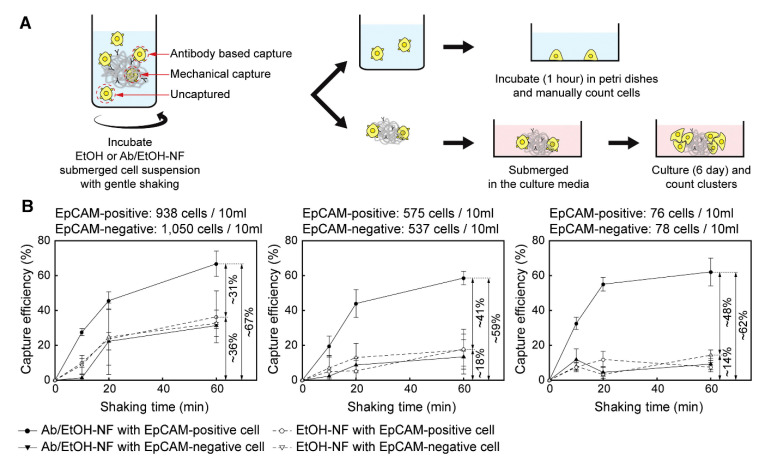
Selective capture capacity of Ab/EtOH-NF. (**A**) Schematic of overall experimental procedure. (**B**) Selective capture capacity of Ab/EtOH-NF with EpCAM-positive cell and EpCAM-negative cell with ~1000, ~500 and ~100 cells per 10 mL (*n* = 3). Reproduced with permission from [58]. Copyright Elsevier, 2017.

PSMA can also be used with other biomolecules; Lee et al. and Yoon et al. used PSMA fiber mats to bind streptavidin to the polymer surface in their development of biosensors. The reactions use phosphate buffered saline (PBS) as the buffer medium at pH 7.4. Though loading was not quantified, both works used fluorescence imaging to show uniform coating of desired materials along the length of the fiber. Lee et al. used the streptavidin immobilized biotin-tagged aptamers for selective capture of thrombin with high specificity and sensitivity (10 pM) [39]. While Yoon et al. attached biotinylated antibodies to the immobilized streptavidin for the selective capture and three-dimensional culture of EpCAM-positive cells in whole blood (Figure 6A,B) [58].

Direct immobilization is the easiest immobilization technique as no surface modification or crosslinker is needed. Works done with enzymes show greater pH and temperature tolerance compared to the free enzyme. This increased tolerance is commonly attributed to conformation of desired enzyme being stabilized and a reduction in extensional deformation of the enzyme. Researchers have also noted a drastic increase in the storage stability and reusability of enzymes immobilized with this technique. Additionally, this technique has been shown to work with other biomolecules such as aptamers. Unfortunately, this technique only works for a few select polymers that contain an epoxide group, limiting its use. Though researchers have grafted epoxide containing groups to polymers, these systems do not result in a similar pH tolerance, showing mainly only a temperature tolerance. 

## 3. Direct Immobilization after Surface Modification

**Figure 7:**
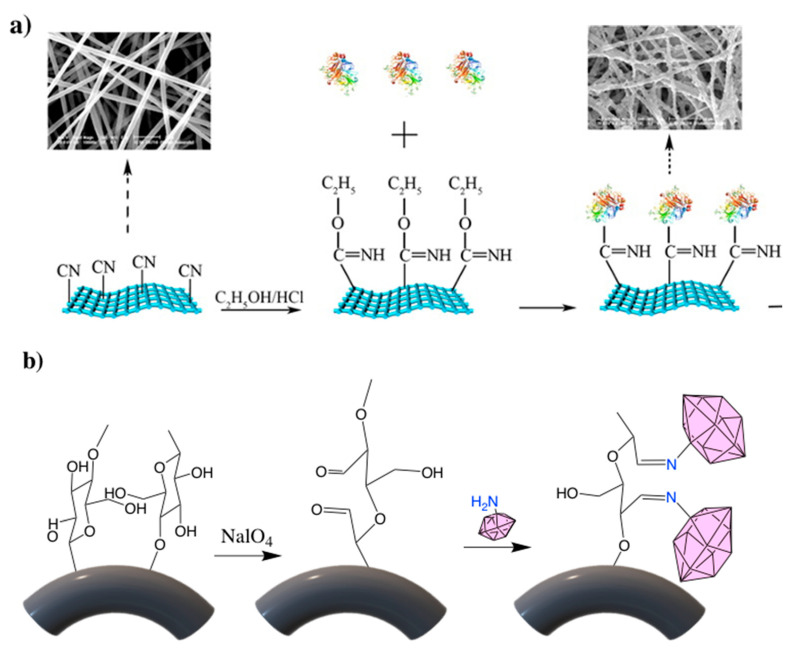
(**a**) Schematic of surface modification of PAN nanofibers using amidination reaction. Reproduced with permission from [67]. Copyright American Chemical Society, 2013. (**b**) Schematic of cellulose derivatives using sodium periodate.

If the desired nanofiber substrate does not contain a strong electrophilic group such as the epoxides previously discussed, surface modifications can be done to introduce desired functional groups to the fiber surface. Polyacrylonitrile (PAN) is a widely used polymer in nonwoven mats due to its good physical characteristics and ease of electrospinning [59,60]. For biomolecule immobilization, PAN is utilized for its mechanical strength and high thermal resistance [61]. However, due to the inertness and hydrophobicity of the acrylonitrile monomer, functional groups must be introduced to the polymer surface through an amidination reaction. Amidination reactions result in imidoester functional groups which react with primary amines to form amidine bonds. This reaction is a derivative of the Pinner synthesis first done by Pinner and Klein in 1877 [62]. In a Pinner reaction a partial solvolysis of a nitrile yields a carboximidate group. Handa et al. were the first to publish this method for use with PAN in 1982 with the immobilization of glucoamylase onto granular polyacrylonitrile [63,64]. For the amidination of PAN nanofibers, the membrane is placed in methanol and dry HCl is pumped through introducing the desired functional group (-OC5H5) to the surface. The resultant carboximidate group then reacts with the amine groups in the enzyme to covalently bond the enzyme to the fiber surface (Figure 7a). The immobilization of the desired enzyme is typically done between a pH of 4.5 and 7 depending on the enzyme used. Researchers who wished to immobilize various lipase enzymes used a neutral pH in phosphate buffer solution [61,65,66]. After immobilization, the lipase enzymes maintained between 79 and 87.5% of the free enzyme activity and displayed superior pH and thermal stabilities. Researchers noted using this direct immobilization method with *Candida rugosa* lipase resulted in better enzyme-membrane interaction noted by the low percentage of increase in K_m_ value after enzyme immobilization. Using a different lipase, *Pseudomonas cepacia* lipase, Li et al. showed a 30% increase in K_m_ using the same technique and PAN nanofiber as the substrate; a slightly lower activity retention is also noted [65]. This indicates the biomolecule-membrane interaction is unique for each case. Xu et al. used a slightly lower pH (6.0) to immobilize laccase (72% activity retention) for 2,4,6-trichlorophenol removal resulting in 87% removal efficiency in 4 h versus 50% for free laccase [67]. The immobilization of laccase resulted in a 60% increase in K_m_ compared to free laccase, a similar value of increase seen by Li et al. in their lipase immobilization study. Hung et al. used an acetic acid solution buffered at 4.5 in order to covalently bond cellulase to the PAN nanofiber surface with 86% activity retention [68]. Comparing the amidination method of cellulase immobilization to glutaraldehyde crosslinking a similar activity retention is noted, indicating the immobilization technique might not be significant for this enzyme. 

This technique demonstrates direct immobilization onto surface functionalized PAN is a viable reaction to be explored. Researchers were able to maintain 70–90% of the initial enzyme activity, while also enabling excellent temperature and storage stability. This is in stark contrast to the previous section involving cyclic groups which saw activity retention values between 2 and 20%. However, amidination is restricted to one particular polymer, PAN, which limits its overall use. Although Jain et al. [69] speculated that the amidination reaction could be used to attach antibodies or other biomolecules to PAN, no literature documenting those reactions was found. Since PAN has poor biocompatibility, the usefulness of attaching biomolecules is limited.

Similar to PAN, the surface of cellulose and cellulose derivatives can also be functionalized to make them more responsive to biomolecule reaction. Sodium periodate (NaIO_4_) has been used to activate the hydroxyl groups of regenerated cellulose-based nanofibers to create aldehyde groups that can be used as binding sites for immobilization (Figure 7b). During this reaction, there is a selective cleavage of the C2-C3 bond resulting in the formation of two aldehyde groups [70]. Prolonged exposure to the oxidation reaction can lead to gradual breaking of the polymer chain resulting in degradation of the cellulose molecules and loss of mechanical strength [71,72]. Nanofibers are especially susceptible to this due to their high surface area to mass ratio. Ma et al. observed a ~90% reduction in tensile strength with increase in oxidation time though immobilized bovine serum albumin (BSA) increased drastically (1 mg/g fiber to 40 mg/g fiber) [71]. On the other hand, Huang et al. also noted a decrease in enzyme loading after a longer reaction time; they attributed this decline to the newly formed aldehyde groups becoming gradually oxidized by NaIO_4_ reducing the amount of aldehyde groups that can bind with the target enzyme [72]. Comparing *Candida rugosa* lipase loading using this method to lipase loading onto amidinated PAN shows a drastic reduction in activity, 29.6 U/g vs. 32.23 U/mg respectively. This is most likely due to the previously mentioned oxidation of the aldehyde groups to carboxyl groups hindering the reaction and limiting enzyme activity. This method was also used to bind proteins, but low binding efficiency to the desired IgG protein was noted [71]. Researchers observed only one IgG molecule was captured for every 30 binding sites. This low binding capacity was attributed to loss of lysine groups during immobilization and steric hindrance of the large IgG molecule.

Though direct covalent bonding onto nanofibers using surface modification is a relatively easy and efficient process, there are drawbacks. Both amidination and NaIO_4_ treatment can cause damage to the nanofiber fiber substrate. Special care must be taken to optimize functional group activation while maintaining the mechanical integrity of the membrane. In addition, as previously mentioned, all techniques discussed in this section are particular to a specific polymer. In order to be applicable to more polymers, a more widely applicable covalent binding or cross linker is needed. 

## 4. EDC/NHS

One of the most commonly used crosslinkers is 1-ethyl-3-(3-dimethylaminopropyl)carbodiimide (EDC), a water soluble carbodiimide which will react with the carboxyl groups on the nanofiber substrate [53]. The reaction between the carboxyl groups and EDC results in an active *O*-acylisourea intermediate which can be easily displaced by a nucleophilic attack from primary amino groups of the targeted material. Ghasemi-Mobarakeh et al. first hydrolyzed the surface of poly(ε-caprolactone) (PCL) fibers to induce the needed -COOH groups for Matrigel immobilization using EDC [73]. Results noted an increase in cell proliferation and cell surface contact area that was not noted by simply mixing Matrigel into the electrospinning solution. Alternatively, Choi et al. chose an amine-group-rich block copolymer, PCL-PEG-NH_2_, as the substrate for immobilization and activated the -COOH groups of recombinant human epidermal growth factor (rhEGF) using EDC to then bind to the amine-rich polymer nanofiber. This is an interesting approach as researchers usually choose to activate the nanofiber substrate and not the enzyme. Analogous to Ghasemi-Mobarakeh et al., Choi et al. also noted an improvement in wound healing with covalently bonded rhEGF compared to simply mixing in the protein [74]. 

**Figure 8:**
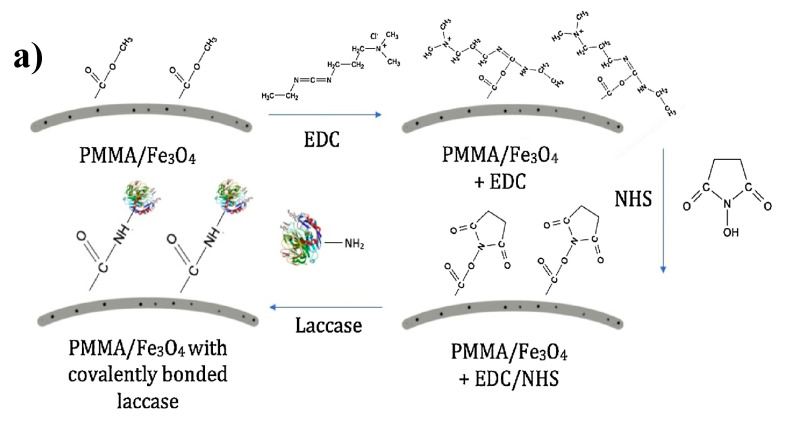

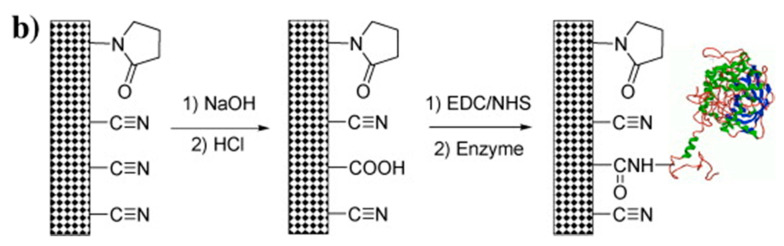
Schematic of EDC/NHS reactions without (**a**) and with (**b**) surface functionalization. Reproduced with permission from [83,88]. Copyright Elsevier, 2019, 2008.

Unfortunately, there are a variety of undesirable *O*-acylisourea intermediates that can form and reduce the reaction yield [75]. These intermediates can include *N*-acylurea [76] which can also rearrange to a racemized version, 5(4*H*)-oxazolones, making portions of the molecule inactive [77]. Because of this, trapping agents are coupled with EDC to reduce side reactions. The trapping agent usually used for applications involving the immobilization of large molecules is N-hydroxysuccinimide (NHS) [78,79]. NHS rapidly reacts with the *O*-acylisourea intermediate creating reactive esters, drastically reducing the side reactions, and in turn, improving reaction yield. The NHS ester will then react with the amine on biological agents by covalent attachment of an acyl group to the nucleophile with the release of the NHS leaving group [80]. This EDS/NHS reaction is commonly referred to as a zero-length crosslinking reaction because the crosslinkers catalyze directional bonding between the macromolecules and the polymer but are not present in the final result (Figure 8a) [81].

Depending on the polymer used, the surface of the electrospun membrane can be activated directly in a one step process or a two-step process; with the two-step process requiring the introduction of a functional group before surface activation using EDC/NHS can be performed. Carboxyl groups containing polymers such as polylactic acid (PLA) [82], poly(methyl methacrylate) (PMMA) [34,41,83] and poly(m-anthranilic acid) (P3ANA) [84,85] are commonly used without the need for initial surface treatment. Tseng et al. [41] electrospun nylon-6/PSBMA/PAA fibrous mats and then used EDC/NHS to activate the fiber surface. The activated fiber mat was then used to immobilize streptavidin and then a biotinylated anti-EpCAM antibody. Researchers noted the new circulating tumor cell (CTC) capture platform was able to reliably detect and capture cells in blood containing artificially low CTC counts. Zhao et al. also noted similar specific cancer cell capture using hyaluronic acid-modified random or aligned PLA fibrous mat in capturing CD44 receptor-overexpressing cancer cells [82]. If the bulk polymer does not contain carboxyl groups, researchers can incorporate carboxylic group elements such as multiwall carbon nanotubes (MWCNTs) in order to facilitate surface activation [86]. Incorporating a carboxyl group containing polymer such as poly(methyl vinyl ether-alt-maleic anhydride) has also been explored by Matlock-Colangelo et al. to activate polyvinyl alcohol (PVA) nanofibers for immobilizing anti- *Escherichia coli* antibodies [87]. Though this method is commonly used with aptamers and antibodies, Jankowska et al. immobilized laccase onto PMMA/PANI using EDC/NHS noting 89% of relative activity compared to free laccase [34]. Compared to adsorbed laccase, researchers noted far superior pH, temperature and storage stability. 

If a polymer does not contain an -NH_2_ or a -COOH group, surface treatments must be done to introduce those groups to the nanofiber surface. This can be done through hydrolysis of the fiber surface [89], plasma treatment [90,91], dip coating [92], and grafting onto the base fiber [93]. Hydrolysis of the fiber surface is commonly done by immersing various polymers such as PAN, its various copolymers, polyhydroxyalkanoate, and PCL in sodium hydroxide (NaOH). PAN based fiber hydrolysis results in the partial conversion of nitrile groups (C≡N) into carboxyl and amine groups which are then activated by EDC/NHS and further used in the immobilization of enzymes (Figure 8b) such as lipase and redoxase [88,94,95]. Chauhan et al. immobilized Anti-VD by first partially hydrolyzing the PAN fiber surface followed by EDC-NHS activation to create Fe_3_O_4_-PANnFs/ITO electrodes for a highly sensitive and selective electrochemical immunosensor for the detection of vitamin D [96]. Treatment of polyhydroxyalkanoate (PHB) fibers with NaOH results in breakage of the ester linkage and formation of carboxyl groups when the ester groups of the polymer chain react with the hydroxide anion [97]. Due to good biocompatibility, PHB is used in systems utilizing the polymer membrane as scaffolds. Masaeli et al. functionalized hydrolyzed PHB nanofibers with collagen and peptides thereby enhancing metabolic activity and proliferation while maintaining neural gene expression [98]. 

Various gases have been used for plasma surface functionalization including oxygen, carbon dioxide and air. Mahmoudifard et al. [99] and Khademi et al. [100] both used oxygen to create the appropriate functional groups on the surface of polyethersulfone. Mahmoudifard et al. noted a uniform coating of EDC/NHS on the plasma treated fiber surface while most of the EDC/NHS washed away from the non-plasma treated fiber surface. Both researchers also found plasma treatment greatly improved the hydrophilicity of the fibers. A similar increase in wettability was seen by Heidari-Keshel et al. after CO_2_ plasma treatment of PHB nanofibers improving collagen immobilization on the fiber surface [101]. Cellular study showed the modified fibers would work well as a tissue scaffold showing better adhesion, growth and viability of Schwann cells in the collagen crosslinked nanofibrous mat compared to the other samples. Rivero et al. also saw promising results upon immobilizing 14-3-3ε proteins onto hydrolyzed PCL nanofibers [102]. Modification of the fiber mats increased cell proliferation from 85% for neat PCL to 105% in PCL-nHA/protein. Researchers also noted a 4% increase in cell adhesion compared to the neat fiber. 

In addition, a -COOH containing polymer can be grafted onto the base polymer using techniques such as atom transfer radical polymerization [103,104]. Sun et al. grafted poly(carboxybetaine methacrylate) (pCBMA) spacer arms onto chitosan nanofibers, decreasing non-specific cell adhesion and improving antifouling properties while introducing carboxyl groups to the surface in order to utilize EDC/NHS coupling for aptamer immobilization [103]. In addition to introducing new advantageous properties to the fiber surface, the addition of polymer spacer arms can minimize the steric hindrance between the tethered enzyme and the nanofiber mat attributed to some of the drawbacks of covalent bonding such as lowered activity due to restricted conformational changes and decreased access to active sites [105].

**Figure 9:**
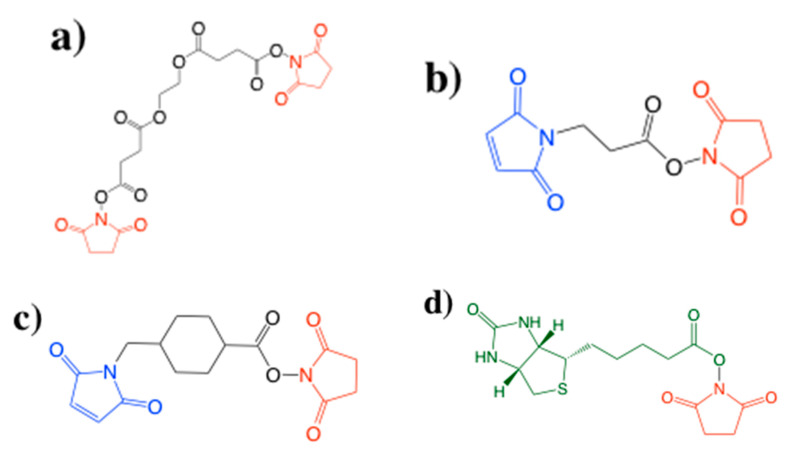
Chemical structures of NHS derivative crosslinkers (**a**) EGS; (**b**) NHS-Mal; (**c**) SMCC; (**d**) NHS-Biotin.

If amine groups are available on the surface of the fibers, whether induced or naturally occurring, an NHS or NHS-malemide compound can be used without the use of EDC. Typically, the use of these compounds does not result in a zero-length spacer arm covalent bond between polymer and biomolecule because the two molecules are tethered together with a spacer polymer. Kim et al. used an NHS homobifunctional crosslinker, ethylene glycol-bis(sulfosuccinimidyl succinate) (EGS) (Figure 9a), to activate both the amine groups in the PCL/PLGA-b-PEG-NH_2_ diblock copolymer nanofiber mat and amine groups of the lysozyme enzyme [106]. Varying the surface available amine groups, researchers were able to achieve a 58% conjugation yield of immobilized lysozyme onto the fiber substrate. Interestingly the highest conjugation yield did not arise from the fiber mat with the highest percentage of amine groups. Researchers hypothesized there was a diffusion limitation caused by the high packing of nanofibers. Favorable in more neutral to basic conditions (pH 7–9), the NHS-maleimide crosslinker will react with the amine in the fiber on the NHS end. While the maleimide will react with thiol groups in the biomolecule to covalently binding the two structures together [107]. Xiao et al. used 3-(maleimido) propionic acid N-hydroxysuccinimide ester (NHS-Mal) (Figure 9b) to immobilize DNA aptamers onto PEI/poly(vinyl alcohol) (PVA) nanofibers [108]. The covalently bonded aptamer was able to capture CTCs with 87% efficiency and nondestructive CTC release of 91%. Park et al. used succinimidyl 4-(N-maleimidomethyl)cyclohexane-1-carboxylate (SMCC) (Figure 9c) for surface activation to covalently bond bone morphogenetic protein-2 (rhBMP-2) onto the chitosan nanofibers [109]. Similar to NHS-Mal, SMCC contains NHS and malemide reactive groups on opposing sides of the polymer chain ligand. However, unlike Xiao et al., researchers saw an increase in amount of immobilized rhBMP-2 with increasing crosslinker concentration until amine groups present on chitosan were fully saturated. Comparing the performance of adsorbed rhBMP-2 and immobilized rhBMP-2, the adsorbed rhBMP-2 membrane showed better initial cell attachment, but the conjugated rhBMP-2 membrane showed much better cell proliferation as time progressed. Instead of activating the fiber surface and then covalently bonding the biomolecule to the surface, Yang et al. used a biotin-NHS (Figure 9d) coupled conjugate as part of their aptamer sensor [38]. The surface of PVA/polyethyleneimine (PEI) was coated with 3-aminopropyltriethoxysilane (APTES), an aminosilane providing a uniform coating of amine groups onto the fiber surface which can react with NHS to bind the conjugate to the fiber surface. Immersing the biotin functionalized fibers in QDs coated with streptavidin (QDs@SA) solution followed by imaging with fluorescence microscopy showed uniform bright red fluorescence across the fiber mat confirming uniform biomolecule attachment. 

Unlike the previously mentioned reactions, the use of the EDC and NHS crosslinkers are not restricted to a particular polymer. Instead the polymer nanofiber membrane simply needs to have suitable -NH_2_ or -COOH functional groups to facilitate functionalization. As mentioned, these functional groups can be a natural feature of the polymer or can be added through an initial surface functionalization step. In the case of -COOH introduction to the fiber surface, there is not only an increase in the amount of available activation sites but also an increase in surface hydrophilicity which also benefits biomolecule immobilization. After enzyme immobilization using EDC/NHS, researchers observe ~40% specific activity retention which is about half the activity retention observed with amidination and other direct immobilization methods. Because of this EDC/NHS is primarily used with other biomolecules such as aptamers and antibodies. Though researchers typically do not report data on percent loading of the biomolecules onto the nanofibers, fluorescence images usually show uniform coating. These aptamers and antibodies are usually used for selective capture of cells and proteins. Immobilizing biomolecules using EDC/NHS shows superior selectivity and specific cell capture compared to the neat fibers as well as better cell proliferation and growth. 

## 5. CDI

CDI (1,1′-carbonyldiimidazole), (C_3_H_3_N_2_)_2_CO, is an organic compound used in the coupling of amino acids for peptide synthesis first discovered in 1957 [110]. It is a highly reactive carboxylating agent with two acyl imidazole leaving groups which can activate carboxylic and hydroxyl groups for conjugating nucleophiles [53]. The reaction mechanisms for each functional group are similar but not the same. In the case of carboxylic groups, the CDI will function as a zero-length crosslinker, similarly to EDC. CDI activates the carboxylic group resulting in the formation of an acyl imidazole intermediate and the liberation of CO_2_ and imidazole. The carboxylate will then react with primary amines to form amide bonds. However, in the case of hydroxyl groups a one carbon spacer is formed [111]. One of the CDI reactive groups will react with the hydroxyl groups on the surface of the nanofiber forming an imidazolyl carbamate intermediate. This highly reactive intermediate will then couple with primary amines in enzymes and biomolecules to form a carbamate linkage [112]. Activating hydroxyl-groups containing polymer nanofibers is the most common usage of CDI in the process of covalently binding a biomolecule, typically enzymes, to nanofibers. Unlike EDC, a highly reactive intermediate is not competing in the amine-crosslinker reaction, allowing for higher reaction yields without the need of a trapping agent. CDI reactions are done in an inert nitrogen environment with anhydrous tetrahydrofuran (THF). PVA and PAA are the most commonly used hydroxyl group containing polymers associated with the CDI crosslinker [113,114,115,116]. 

**Figure 10:**
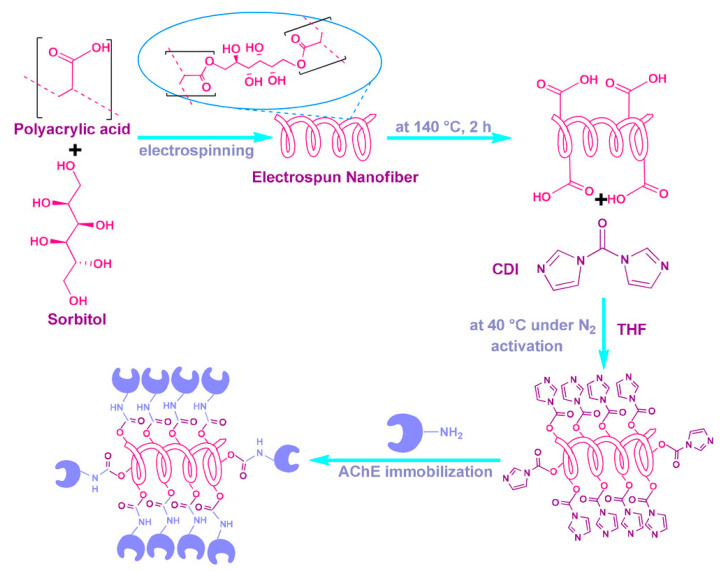
Schematic illustration of immobilization of AChE on electrospun nanofiber membrane. Reproduced with permission from [117]. Copyright John Wiley & Sons, Inc., 2018.

Using this method researchers are able to improve the thermal, pH, and storage stability of the enzymes while retaining a high percentage of the enzyme activity compared to the enzyme in its free state. Çakıroğlu et al. saw that acetylcholinesterase immobilized nanofibers (Figure 10) retain 70% of their initial activity after 60 days, while the free enzyme had lost all activity [117]. In addition, Xu et al. noted the immobilization of HRP onto PVA/PAA/SiO_2_ nanofibers yielded higher loading and activity retention compared to some reported results [3]. This increase in HRP loading is attributed to abundant -OH groups present on the backbone of the PVA/PAA nanofibers which provide more sites of crosslinking. These HRP values were compared to immobilization of HRP onto beads which have lower available surface area per volume compared to nanofibers [118,119]. Immobilized HRP retained 81% of its specific activity, which is higher than what was seen for other nanofiber supports. Researchers credited this to the biocompatible characteristics of the polymer used. In another work done by Xu et al. [3], researchers used Fe_3_O_4_ instead of SiO_2_ in their nanofiber matrix and noted a 85% specific activity retention. This slight increase is credited to improvement of the physical and chemical properties of the membrane by incorporating Fe_3_O_4_. 

CDI appears to be a good crosslinker for activating the surface of the hydroxyl group containing nanofibers. Enzymes retained over 80% of their specific activity which is similar to what was seen using the direct immobilization techniques in Section 3. Compared to the enzyme immobilization using EDC/NHS, another zero-length crosslinker, CDI has double the activity retention. Though CDI has only been used so far for hydroxyl-group containing polymers such as PAA, its use can be expanded to carboxylic-group polymers based on its reaction mechanism. This would allow a greater variety of nanofibers to be used as the base substrate such as PMMA and PLA. 

## 6. Glutaraldehyde

**Figure 11:**
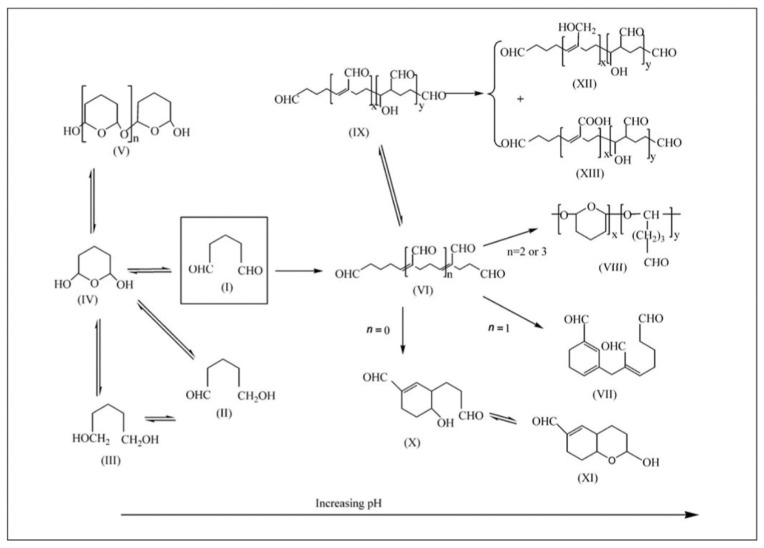
Summary of the possible forms of glutaraldehyde in aqueous solution. Reproduced with permission from [125]. Copyright Future Science Ltd., 2004.

Glutaraldehyde (C_5_H_8_O_2_) is a saturated dialdehyde commonly used as a chemical crosslinker in a variety of applications such as microscopy [120,121], the tanning industry [122], biomolecule immobilization [123,124] and many others. Though it is widely used, the exact crosslinker for the immobilization of amine-containing biomolecules and the mechanism for its reaction with materials are not known because its exact structure in solution is under fierce debate [125]. Depending on pH and aqueous media, it is believed glutaraldehyde can be present in its monomer form, a dimer, trimer or as a polymer (Figure 11). Due to uncertainties about its polymer size and structure, it is impossible to know the exact nature of the conjugates formed. In addition, for successful immobilization of biomolecules such as enzymes using glutaraldehyde a rigid control of reaction conditions is needed. Researchers must consider pH, concentration, temperature, reaction times, et cetera. Even when considering all these components, it can be difficult to get reproducible results. In addition to amine groups, glutaraldehyde can also react with hydroxyl groups to form acetal bonds [126]. It is widely used for the crosslinking of PVA under acidic conditions for this purpose [127,128]. 

**Figure 12:**
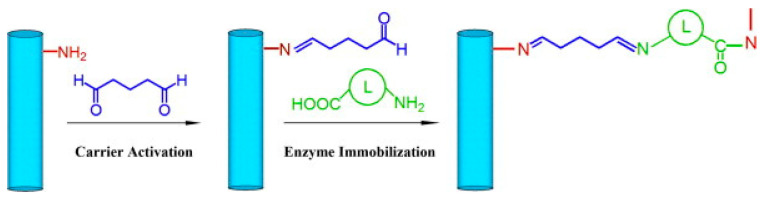
Illustration of carrier activation and enzyme immobilization using glutaraldehyde. Reproduced with permission from [137]. Copyright Elsevier, 2013.

Similar to EDC/NHS, glutaraldehyde can be used as a crosslinker with or without surface treatment depending on the functional groups present on the fiber. Like NHS, glutaraldehyde reacts with amine rich substrates including naturally occurring amine containing polymers (Figure 12) such as chitosan [129,130], silk [131] and zein [132,133] as well as thermoplastics such as polyamides [134]. Nylon 6,6 electrospun fibers were activated by glutaraldehyde for the immobilization of laccase of enzymatic degradation of ginkgolic acid by Chen et al., improving enzyme stability and reusability [135]. Compared to immobilization on a nylon pellet, researchers noted a significantly higher Km value, indicating enhanced surface affinity due to decreasing the size of the immobilizing platform.

**Figure 13:**
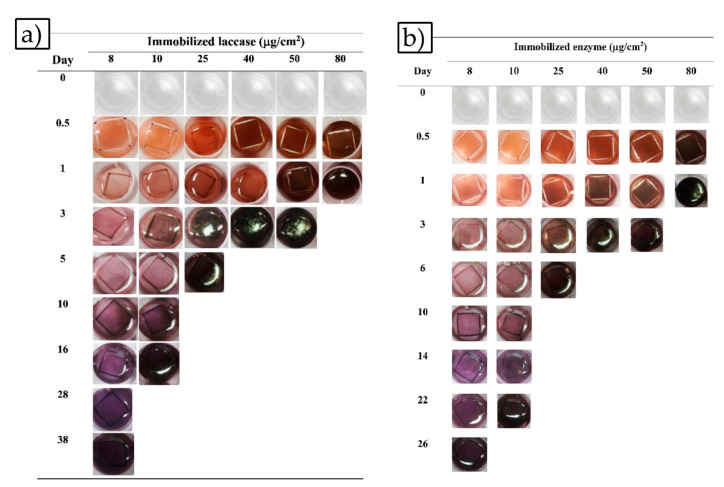
Color change of (**a**) ceCPL (8–80 μg/cm^2^) stored in a 4 °C showcase refrigerator and (**b**) ceZL (8–80 μg/cm^2^) reacted with guaiacol at 4 °C. (**a**) Reproduced with permission from [143], Copyright Elsevier, 2020. (**b**) Reproduced with permission from [132]. Copyright Elsevier, 2020.

Chitosan is the most widely used polymer when using glutaraldehyde as a crosslinker without surface treatment. It is usually coupled with polymers such as nylon 6 [136], PVA [134,137], PEO [138], or PVP to facilitate the spinning process. Though chitosan is valued for its biodegradability and non-toxicity, it is difficult to electrospin due to its polycationic nature in solution, rigid chemical structure, strong hydrogen bonds and repulsive forces between ionic groups on the polymer backbone [139,140,141,142]. Jhaung et al. produced time-temperature indicators for food quality monitoring by immobilizing laccase onto chitosan/PVA fibers [143]. The immobilized laccase catalyzes the oxidation of phenolic compounds showing visual and color changes from transparent to deep brown or deep purple-brown (Figure 13a). Immobilized laccase maintained 70% of its relative activity using glutaraldehyde as a crosslinker and also retained 94.53% residual activity after 10 days of storage. These values are in line with residual activity values reported by other researchers using other techniques [34,135]. Xu et al. [144] found the introduction of MWCNTs into their chitosan/PVA fibers improved enzyme loading, activity retention, pH stability, thermal stability, operational stability, and storage stability by providing enhanced electrical conductivity and biocompatible microenvironments similar to the effect seen by incorporating Fe_3_O_4_ into PVA/PAA matrix. In this study, researchers saw a 76% activity retention of laccase under optimal conditions, agreeing with Jhaung et al.’s findings. PVP/chitosan/reduced graphene oxide (rGO) nanofibers were electrospun and activated with glutaraldehyde by Pavinatto et al. for immobilizing laccase [145]. Glutaraldehyde can crosslink with both the -NH2 groups in chitosan and the -OH groups in the graphene sheets creating multiple avenues of enzyme immobilization. The developed biosensor involving the immobilized nanofiber showed excellent biosensing behavior with a very low limit of detection of 0.15 pmol L^−1^. Xu et al. chose to purchase chitosan fibers, allowing them to bypass the difficulty of electrospinning pure chitosan nanofibers to serve as their substrate for polygalacturonase immobilization [146]. 

Lee et al. utilized another naturally occurring material, silk, to immobilize α-chymotrypsin [131]. Results indicate immobilization improved enzyme stability at ambient temperatures but not at elevated temperatures which was attributed to restriction of conformation change due to limited enzyme attachment on the nanofiber. In addition to their previously mentioned work with chitosan/PVA [143], Jhuang et al. have also used zein as a nanofiber substrate to immobilize laccase for TTI development [132]. Zein is a plant protein; unlike chitosan, the researchers were able to electrospin zein alone without the need for a copolymer. The activation energy, E_a_, of coloration was lower for this experiment (26.28 kJ/mol) vs 40.9 ± 2.8 kJ/mol for chitosan/PVA fibers both with 80 μg/cm^2^ loading of laccase though their color response tests were similar (Figure 13b).

Researchers can also take advantage of glutaraldehyde reacting with hydroxyl groups. Doğaç et al. immobilized lipase onto PVA/alginate fibers utilizing the hydroxyl groups of the polymer, which saw a higher enzyme loading compared to the PEO/alginate fibers being used [147]. Similarly, Işik et al. immobilized lipase onto PVA/Zn^2+^ metal composite nanofibers which resulted in improved reusability, 80% after 15 uses; a 30% improvement over Doğaç et al.’s result which saw 50% after 14 uses [148]. This improvement is contributed to secondary interactions between the enzyme and nanofiber. Sathishkumar et al. took advantage of the numerous hydroxyl sites on electrospun cellulose nanofibers to immobilize laccase for loading to up to 85% dye discoloration after 5 cycles [149]. 

As previously mentioned, nylon can be activated by glutaraldehyde directly, however some researchers chose to first hydrolyze the nylon nanofibers before activation. Using 3 M HCl for 2 h is the optimal reaction conditions for this reaction, leading to maximum enzyme immobilization without dissolving the nanofiber. Hydrolysis of nylon fibers gives rise to more -NH_2_ groups on the fiber surface which in turn leads to high enzyme loading [150,151]. Fatarella et al. attained 71% laccase immobilization on hydrolyzed nylon 6 nanofibers compared to 34.5% onto neat nylon 5 fibers under the same reaction conditions [4]. Harir et al. obtained a similar enzyme loading, 82%, of tyrosinase onto nylon 6 nanofibers noting increased storage stability [152]. Wang et al. subjected the nylon 6,6 nanofibers to UV-zone treatment in order to also introduce primary and secondary amines to the fiber surface [47]. Researchers noted a 250% increase in chymotrypsin immobilization on the nylon nanofibers compared to nylon film treated in the same manner. In addition, results suggested immobilization onto nanofibers mimics free enzyme activity more than that of microscale substrates while providing increased temperature and pH stability.

**Figure 14:**
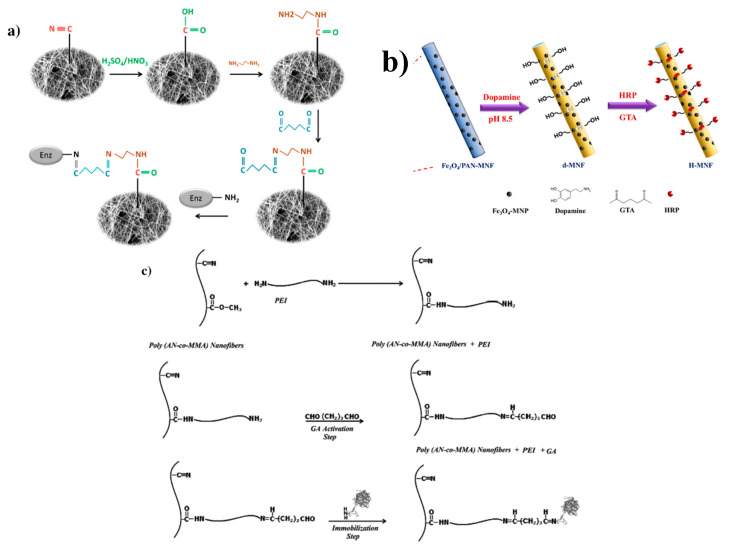
Schematic of the various surface functionalizations that can be performed on nanofibers before surface activation using glutaraldehyde. (**a**) Surface coating; (**b**) Treatment with NaOH and HNO_3_/H_2_SO_4_; (**c**) polymer grafting. Reproduced with permission from [35], Copyright American Chemical Society, 2017. Reproduced from [160], Copyright Elsevier, 2019. Reproduced from [159], Copyright John Wiley & Sons, Inc., 2012.

The cyano groups of PAN have been addressed in a variety of ways including plasma treatment and treatment with NaOH. Taheran et al. reacted PAN with NaOH similarly to work done with EDC/NHS, to introduce -COOH groups to the fiber surface. The -COOH groups were then reacted with ethylenediamine to introduce the needed amine groups for glutaraldehyde activation (Figure 14a) [35]. Xu et al. used a combination of NaOH and absolute ethyl alcohol to convert the nitrile groups to amide groups which can then freely react with the crosslinker to also immobilize laccase [153]. Similarly, Farzin et al. used chemical modification using hydroxylamine to convert the nitrile groups of PAN to amidoxime groups [154,155]. The amidoxime groups reacted with the crosslinker which was used to bind an aptamer to the surface for the use as an electrochemical biosensor for the sensitive detection of CA-125 cancer cells, having LOD’s levels below or on par with other CA-125 detection literature. In contrast, Mahmoudifard et al. used ammonia plasma to create amine groups on the PAN surface by both reduction of the nitrile groups via hydrogen fragment generated through the decomposition of plasma and also direct incorporation of amine fragments that exist in the plasma gas [156]. Researchers noted a significant enhancement in enzyme-linked immunosorbent assay (ELISA) signal compared with hydrophobic physical adsorption. 

A variety of other methods can be employed in order to introduce the desired amine group into the surface of fibers lacking this functional group [157]. Temoçin et al. converted the amide groups of PVA/polyacrylamide (PAAm) to amine groups via the Hofmann degradation reaction using sodium hypochlorite (NaOCl) followed by NaOH [158]. The altered fibers were activated with glutaraldehyde then used to immobilize HRP which retained 63% of its initial activity; this value is lower than values reported by Xu et al. [113] using CDI as the activation agent which resulted in 81% activity retention. However, the most used method was simply coating the base nanofiber with a polymer containing the desired functional group. In order to introduce amine groups, researchers have used PEI, a highly cationic polymer with numerous amine groups on the polymer chain or a -OH containing group such as dopamine. El-Aassar et al. showed that an increase in PEI concentration resulted in an increase of catalytic activity and retention of activity of immobilized β-galactosidase [159]. Both Xu et al. and Li et al. sought to immobilize HRP, using glutaraldehyde as an activator but using different polymers (PEI vs dopamine) to coat the base fiber to degrade phenol. Li et al. used dopamine which introduced hydroxyl groups to the surface of Fe_3_O_4_ /PAN magnetic nanofibers (MNFs) (Figure 14b); after HRP immobilization the researchers were able to remove 85.2% of phenol at optimal conditions [160]. While Xu et al. utilized PEI to coat poly(methyl methacrylate-co-ethyl acrylate) (PMMA CEA) nanofibers and were able to remove 93% of bisphenol A (BPA) from solution [161]. Laccase immobilization using glutaraldehyde and PEI activated fibers was done by both Koloti et al. [162] (PES base fiber) and El-Aassar et al. (Figure 14c) [163] (poly(acrylonitrile-co-styrene/pyrrole) base fiber), showing enzyme loading and high retention capacity. Koloti et al. reported 88.7% removal of BPA from solution, falling in line with previously mentioned work using surface modified glutaraldehyde fibers. APTES has been used as a surface modifier on cellulose triacetate and PAA nanofibers to introduce amine groups to the fiber surface [164,165]. Vahid Ebadi et al. chose APTES as a surface modifier and glutaraldehyde as the crosslinking agent to immobilize acetylcholinesterase to a nanofiber substrate for the first time maintaining 90% of its original activity after 10 cycles [165]. 

Glutaraldehyde is a versatile crosslinker that can be used to activate a variety of nanofibers that contain amine groups. It is used to immobilize enzymes, antibodies and other biomolecules adding to its versatility. However, because so much is unknown about the state of this crosslinker in solution, it can be difficult to predict the final crosslinked product and achieve reproducible results. This could pose a challenge if using this crosslinker on a large scale for commercial use where reproducibility is important. 

## 7. Combined Techniques

**Figure 15:**
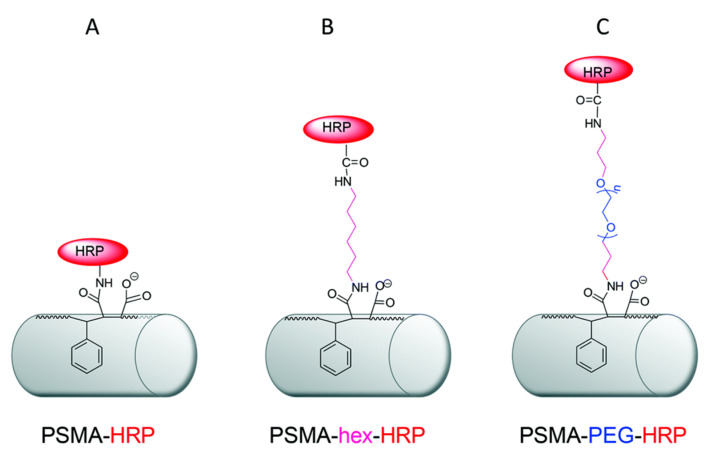
Schematic view of the different HRP functionalized fibers: PSMA-HRP (**A**), PSMA-hex-HRP (**B**), and PSMA-PEG-HRP (**C**). Reproduced with permission from [168]. Copyright Royal Society of Chemistry, 2017.

Though researchers typically utilize only one crosslinker for biomolecule immobilization, some have coupled various crosslinkers and techniques. If a substrate contains both -COOH and -NH_2_ researchers have used both EDC/NHS and glutaraldehyde to achieve maximum biomolecule loading with catalytic activity [166,167]. In addition, Martrou et al. [168] and Dai et al. [169] both noted an increase in biomolecule loading and stability with increasing spacer arm length. Martrou et al. used three modes to immobilize HRP onto PSMA nanofibers of increasing spacer arm length: (1) direct immobilization employing epoxide ring opening (as noted in Section 2), (2) activation with hexamethylenediamine (hexDA) coupled with EDC/NHS, (3) PEG diamine (PEGDA) coupled with EDC/NHS (Figure 15). Results noted activity retention of 2.4%, 19.7% and 34%, respectively. This increase is attributed to both the increase in hydrophilicity due to the spacer arm attachment and the increase in enzyme accessibility to the active sites on the substrate. Dia et al. noted a similar trend using direct immobilization, glutaraldehyde, and HMDA coupled with glutaraldehyde. Researchers noted not only higher enzyme loading, but also higher enzyme thermal stability. This is credited to restriction of conformational transition of the enzyme because of multiple attachments sites to the nanofiber mat, protecting the distortion of the enzyme at high temperature. It is important to consider not only the length of the spacer arm but the nature of the spacer itself. Maryšková et al. used both a (1) GA+HDMA+GA and (2) GA+BSA+GA configuration to immobilize laccase with similar loading [136]. However, the storage stability of the GA+BSA+GA fiber mat is similar to that of the free enzyme, while the GA+HDMA+GA fiber mat had a 26% improvement in residual activity after 14 days. 

## 8. Click Chemistry

Click chemistry, named by Karl Barry Sharpless in 1998, is a broad class of reactions that are characterized by relatively fast, catalyzed reactions, high efficiency, high selectivity and stable reactants [170]. A key advantage of click reactions is that they are bio-orthogonal and produce stable covalent bonds, and therefore, biomolecules can participate in the reactions while retaining their bioactivity [170]. Reactive sites must be introduced onto the biomolecule and subsequently clicked either directly onto the nanofiber backbone or onto a modified nanofiber surface. Various click reactions have previously been demonstrated as a successful immobilization method for cyclodextrin [171,172,173], other saccharides [174,175,176], fluorescent tags [177,178] and PNIPAM brushes [179,180,181]. Since click chemistry is still in the exploratory stage as a technique for functionalizing electrospun nanofibers, the majority of the studies use model biomolecules such as biotin [178,182,183,184,185], polymers [186,187], metallic nanoparticles [188], and various small molecules [189,190]. These models are used to demonstrate the potential of the click method, but they do not have the higher order of structural complexity that biomacromolecules such as antibodies and enzymes possess or the challenge of losing bioactivity due to the biomolecule functionalization step. The click reaction itself is highly selective, but the methods to obtain the clickable biomolecule must also be specific to ensure preservation of bioactivity. Shi et al. attempted to achieve the means to this end with their modified breast cancer biomarker, TSP50. TSP50 does not naturally contain an azide group, so excess azide must first be substituted onto the hydroxyl, amine, or halogen groups on the protein. This reaction is stochastic; the location and degree of azidation was not determined in the study, but the activity of the bound antibodies was confirmed with ELISA assay [191]. More complex techniques to achieve selective conjugation, which involve site-specific labeling of biomacromolecules, have been developed in parallel and discussed in a recent review [192]. 

**Figure 16:**
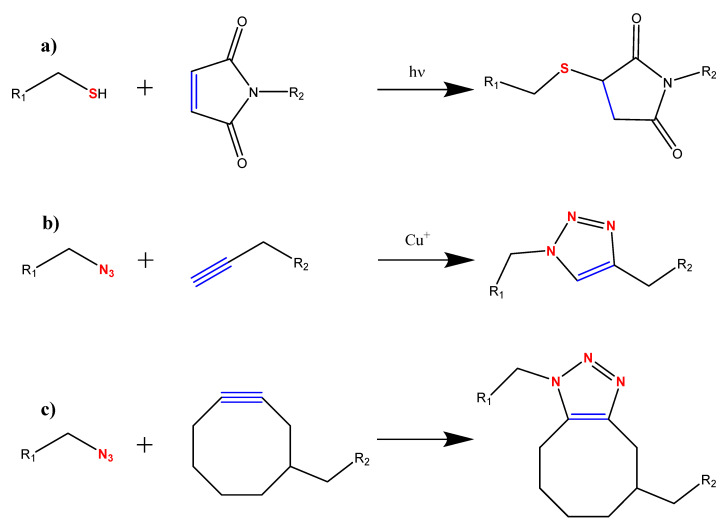
General reaction schemes for (**a**) Radical-mediated thiol-ene click reactions, (**b**) Copper-catalyzed Azide Alkyne Cycloaddition (CuAAC), (**c**) Strain-Promoted Azide Alkyne Cycloaddition (SPAAC). R_1_ and R_2_ can represent either the biomolecule or the nanofiber.

Thiol-ene click reaction (Figure 16a) is a radical-mediated, usually photo-initiated, coupling of alkene and thiol moieties with a well understood reaction mechanism [193]. Thiol-ene click chemistry is a desirable conjugation method for biomolecules because cysteine (amino acid with thiol side chain) naturally occurs in peptide sequences. Limitations of this conjugation are: lack of selectivity when biomolecules contain multiple native cysteine residues, requirement for artificial modification of the biomolecule if it does not naturally possess a cysteine, and loss of bioactivity if the cysteines are within the active site. Song et al. purchased a custom antimicrobial peptide, Cys-KR12, that contained a cysteine attached to the N-terminus of the otherwise thiol-free KR12 peptide and clicked it to modified silk fibroin nanofibers [194]. The N-terminus was chosen for its higher hydrophilicity, compared to the C-terminus, which directly affects the affinity of the biomolecule towards the substrate surface during the click reaction. This allowed for proper orientation of the peptide without the need for a spacer arm. Silk fibroin was electrospun and sequentially prepared by EDC/NHS activation, N-(2-aminoethyl)maleimide (AEM) linker attachment, and finally clicked with Cys-KR12. Electron poor alkenes such as maleimide are slower than electron rich and strained alkenes [193], but the click conjugation was still relatively fast with a reaction time of 4 h. The immobilization density of Cys-KR12 was linearly proportional to the concentration of Cys-KR12 in the reaction solution, and higher density translated to better bioactivity performance. The bioactivities tested included antimicrobial activity (inhibit concentration and longevity), cell proliferation and differentiation, and cytotoxicity. Most notably, the immobilized Cys-KR12 at 200 and 500 μg/mL densities exhibited minimum inhibitory concentrations (MICs) comparable to the soluble Cys-KR12 against four bacteria strains and encouraged cell proliferation where soluble Cys-KR12 exhibited cytotoxicity above 100 μg/mL. Site-specific addition of the cysteine to the peptide proved to be a smart strategy, as all tested concentrations had over 90% yield and the proper peptide conformation was preserved to exhibit the desired antimicrobial activity. 

Alternatively, alkene groups can be incorporated into the biomolecule and click with a thiol-modified nanofiber surface. Chitosan and polycaprolactone (PCL) fibers were electrospun then modified to obtain thiol groups on the surface [195,196]. Chitosan naturally possesses amine groups on the polymer backbone, but PCL required intermediate functionalization steps involving UV/ozone irradiation and aminolysis to prepare the mats for thiol insertion. Thiol concentration as a function of pH and reaction times for UV/ozone irradiation, thiol insertion, and thiol quantification were tested for the corresponding fiber systems. A neutral pH correlated to the highest free thiol on the chitosan fibers. The balance between thiol concentration and morphology preservation was determined to be at 4 min of UV/ozone irradiation, 3 h for thiol insertion, and 3 h for thiol quantification. Both thiol insertion methods achieved uniform distribution of the thiol groups on the surface of the fibers. Both fiber systems were clicked with liposomes decorated with maleimide probes on the liposome surface. Liposomes are biomolecules that are widely used for their capability of drug carriage and local drug release and adaptability of structure and surface. The liposome was also designed with cholesterol and PEG components to aid in the affinity of the liposome to the modified fiber and liposome stability, respectively. Drug release kinetics and bioactivity were tested for both fiber systems. The ability to control the fiber and liposome surface chemistry was the key to the click strategy success in these studies. 

The copper-catalyzed azide-alkyne cycloaddition (CuAAC) click reaction (Figure 16b) obviates the challenge of the biomolecule’s residues participating in the click reaction; it is irreversible and highly selective to only a couple azides and alkynes to form a stable triazole ring [197]. Topological and electronic similarities of the triazole ring to the peptide bond provide an additional advantage to using click chemistry as certain biological activity can be mimicked from the peptide bond without having to worry about the susceptibility of hydrolytic cleaving even under extreme conditions involving denaturants, organic solvents, and acidic or basic conditions [198,199]. As previously stated, Shi et al. aimed to demonstrate the potential of clicking a breast cancer biomarker (TSP50) to L-lactide and 5-methyl-5-propargyloxycarbonyl-1,3-dioxan-2-one copolymer (P(LA–co–MPC)) [191]. The synthetic copolymer contains pendant alkyne groups that were directly clicked with the azide-modified TSP50. ELISA assays with anti-TSP50 and HRP-IgG determined that blocking agents were required to suppress nonspecific binding, and that approximately half of the TSP50 active sites preserved their activity after the blocking step. Further, the specific interaction between anti-TSP50 and immobilized TSP50 was broken with highly acidic buffer (2.2 pH) to elute the anti-TSP50 and regenerate the TSP50 immobilized on the nanofiber membrane. The capture capacity (quantity of captured anti-TSP50 divided by quantity of bound TSP50), elution efficiency, anti-TSP50 activity retention, and immobilized TSP50 regeneration efficiency were ~70, 80, 90, and 75%, respectively. 

Copper is known to be cytotoxic at a threshold concentration, and residual copper absorbed by the nanofiber membranes can cause problems if the intended end-use is biologically oriented. While some of these studies tested for cytotoxicity, another approach, strain promoted azide-alkyne cycloaddition (SPAAC) click reaction (Figure 16c), has been explored to completely circumvent the use of copper. The pendent alkyne in the CuAAC reaction is replaced with a strained alkyne, and the SPAAC reaction rate is typically slower than CuAAC surface reactions [200]. Callahan et al. clicked azide-modified YIGSR peptide onto 4-dibenzocyclooctynol (DIBO)-terminated poly(L-lactide) (PLLA) electrospun nanofibers [201]. End-functional PLLA with targeted high molecular weights and narrow molar mass distributions was achieved by ring-opening polymerization of L-lactide with DIBO as initiator and 1,8-diazabicyclo[5.4.0]undec-7ene (DBU) as catalyst. The YIGSR peptide derivative was synthesized via standard solid-phase FMOC chemistry where the N-terminus was converted with 6-bromohexanoic acid. After purification, the bromide group on Br-YIGSR was substituted with an azide group, purified again, and finally lyophilized. The azide substitution was confirmed by ESI-mass spectra, which determined the molecular weight of the modified peptide to be 789.3 Da. FMOC chemistry ensures that the bromide group is specifically bound to the N terminus of the peptide, but no discussion was made whether the azide also specifically binds to the N terminus or if nonspecific reactions with the amino acid side chains along the peptide were suppressed. The concentration of YIGSR clicked onto the fiber, 4.94 ± 2.76 mg YIGSR/ g fiber, was determined by Lowry assay. While the bioactivity of the immobilized YIGSR was demonstrated with proof-of-concept cell cultures, the bioactivity retention of the immobilized versus free YIGSR was not specifically tested to determine the immobilization strategy’s effectiveness. Further, this metal-free method is acclaimed for circumventing the cytotoxicity challenge, so cell viability studies of SPAAC versus CuAAC clicked YIGSR peptides would verify the practicality of applying SPAAC over CuAAC in subsequent functionalized nanofiber systems.

**Figure 17:**
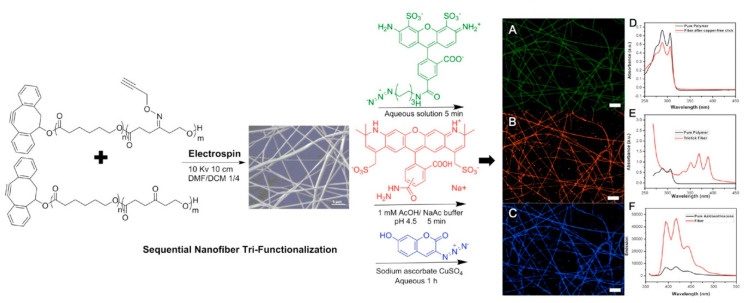
(**A**–**C**) Fluorescence images showing successful sequential trifunctionalization of fibers. The scale bar is 20 μm for all images (**D**–**F**). UV–visible absorption spectra show evidence of the successful copper-free click reaction and copper-catalyzed reaction by the reduced DIBO signal and the appearance of the 9-methyleneazidoanthracene signal at 306 nm and 325–400 nm, respectively. Reproduced with permission from [203]. Copyright American Chemical Society, 2015.

As discussed in Section 7, the bio-orthogonality and selectivity of individual click reactions can be leveraged to specifically immobilize multiple biomolecules onto one nanofiber membrane. Zheng et al. have demonstrated successful dual and tri-click immobilization of biomolecules onto electrospun nanofibers. SPAAC and oxime ligation were conducted in one pot onto nanofibers electrospun from synthetic polymers containing DIBO and ketone groups [202]. The bioactivity of the two immobilized peptides was confirmed with Schwann cell attachment and growth. Separately, SPAAC, oxime ligation, and CuAAC click reactions were performed sequentially onto nanofibers electrospun from synthetic polymers containing DIBO, ketone, and alkyne groups [203]. Importantly, SPAAC was reacted before CuAAC, as CuAAC would click onto both DIBO and alkyne groups whereas SPAAC can only click onto DIBO groups. Three separate fluorescent molecules were first tested to demonstrate the success of the three sequential clicks (Figure 17A–C). The adhesive peptide sequence GRGDS (N3-GRGDS), a tethered calcium-binding dopamine species (NH2O-dopamine), and an osteoinductive BMP-2 peptide sequence (N3-BMP-2 peptide) were chosen as models for proof-of-concept to “triclick” biomolecules onto the previously mentioned synthetic polymer nanofiber. The concentration of GRGDS and BMP-2 peptide on the surface of the nanofibers was calculated after each respective click reaction using the Lowry assay to be 13.1 ± 5.2 μg/mg and 13.2 ± 5.1 μg/mg. The dopamine concentration was calculated to be 16.0 ± 3.2 μg/mg using UV-visible spectroscopy (Figure 17D–F) and a calibration curve of NH2O-dopamine in solution. Both studies highlight the potential of the multi-click nanofiber systems in tissue engineering, but the multiplexing of biomolecules also finds importance in other areas such as ultrafiltration and diagnostic assays.

The most notable advantage of click chemistry is the bio-orthogonality and selectivity of the reactions, but the most notable disadvantage is that, in order to achieve the specific groups on the biomolecule and nanofiber surface, multiple reaction steps must be carried out on the respective units to prepare them for the click reaction. This can translate to custom synthetic polymers, highly involved biomolecule synthesis and modification, pre- or post-fabrication functionalization of the nanofibers, or combinations thereof. Considering this shortcoming, the studies discussed here illustrate that as long as the nanofiber and biomolecule have the complementary click moieties, click chemistry is a promising method to selectively immobilize biomolecules while preserving their bioactivity.

## 9. Future Direction and Conclusions

Some recent studies have shown promise for immobilizing biomolecules onto various substrates other than electrospun nanofibers. Odinolfi et al. grafted a copoly azide crosslinker arm onto nitrocellulose slides (Fast Slide 16-Pad, Whatman, Maidstone, UK), nitrocellulose blotting membranes (BioTrace NT blotting membranes from PALL), and Whatman filter paper 1 before demonstrating peptide co-clicking [204]. Replacing the study’s cellulosic surfaces with cellulosic (cellulose acetate, nitrocellulose, regenerated cellulose, etc.) nanofiber membranes would be facile and the logical next step for increasing the number of available active sites for peptide immobilization. Mou et al. leveraged specific pathogenic bacteria’s ability to bind Cu^2+^ and reduce it to Cu^+^ to design a point-of-care colorimetric sensor [205]. The bacterial reduction of copper triggers the CuAAC click reaction between azide and alkyne functionalized gold nanoparticles that subsequently amass together. The size of the aggregations changes the sample solution from red to blue and allow for rapid visualization of microbial presence in the sample. The bacteria copper binding and internal redox enzyme cascade can be applied to the CuAAC click reactions on the surface of any type of nanofiber by controlling the local concentration of copper so that the membranes do not absorb the residual copper and instead maintain the biocompatibility of the post-click membrane. Feng et al. used UV radiation to break a tetrazole ring to remove N_2_ and insert thiol [206]. Potentially, the PAN nanofiber membrane can first be functionalized by a click reaction that forms the tetrazole ring (first functional molecule or a dye group), then the ring can stably be broken to add an additional functionality while still maintaining the ultra-selectivity of both reactions. The sequential click reactions allow for multifunctionality on a single probe, which can be used for visualization or various other imagined functional systems. Alonso et al. reported the first rapid, efficient, and site-directed immobilization of half immunoglobulin G (hIgG) antibodies on glass or other Si-based surfaces [207]. The disulphide bonds bridging the two halves of the antibody were first reduced to obtain the two halves with the newly exposed thiol groups on each side of the broken bridge. These hIgG were successfully clicked onto alkene-functionalized glass surfaces via UV light-induced thiol–ene click reaction. The half antibodies are critically important in diagnostic assays as antibodies are bulky biomacromolecules, and their immobilization concentration is limited by the monolayer surface area of the nanofiber. Clicking the half antibodies allows for directed orientation and enhanced alignment of the immobilized molecules that improves their response compared to whole antibody microarrays [207]. The half antibody click strategy could be easily applied to the maleimide-modified nanofiber membranes described in Section 4 (EDS/NHS) and Section 8 (Click Chemistry).

All the methods mentioned so far have utilized some form of wet chemistry in order to immobilize biomolecules. However, within the last decade researchers have shown that biomolecules can be immobilized using linker free methods [208,209,210]. One such method is plasma immersion ion implantation (PIII) which modifies the polymer surface enabling covalent immobilization without using linker chemistry. During PIII treatment, the polymer surface is blasted with ions from an ionized gas, with energies ranging from 5 to 20 KV. The energy deposited during the collision of the ions with the polymer surface breaks chemical bonds and causes displacement and excitation of atoms and electrons [211]. The process results in highly reactive chemical groups a few hundred nanometers below the polymer surface; the embedded radicals then slowly diffuse to the fiber surface and become available to form covalent linkages with biomolecules. Kosobrodova et al. found anti-CD34 antibodies formed a uniform layer on the surface of PIII polycarbonate film with most of the antibodies possessing an “active-site-up” orientation which led to greater accessibility of the desired antigen. This technology could easily be used on polytetrafluoroethylene (PTFE), polypyrrole and polycarbonate electrospun fibers for use in microassays and immunosensors.

Though PIII removes the need for wet chemistry for biomolecule immobilization, it does still require low energy plasma which necessitates the use of vacuum chambers, pumping stations and other equipment. Recently Bliek et al. proposed the use of air atmospheric pressure plasma to immobilize tropoelastin onto PTFE foil [212]. The use of dielectric barrier discharge in air at atmospheric pressure eliminated the need for some of the equipment needed for PIII, while still creating radicals to participate in the immobilization reaction. Researchers noted the treated PTFE surface showed significant improvement in cell adhesion and proliferation compared to the untreated surface. Applying this process to PTFE nanofibers would result in even better tropoelastin retention due to the increased surface area of the nanofiber membrane.

These studies implemented novel designs not seen yet in the nanofiber research space. Applying these novel designs to nanofiber surfaces could further expand the outlook for biomolecule immobilized nanofiber membranes while increasing the commercial feasibility of the novel designs.

This review provides an overview of the various methods used to immobilize biomolecules onto electrospun nanofibers. The methods include direct immobilization methods which currently are used primarily with enzymes. Direct immobilization eliminates the need for linkers but limits the researcher on the type of nanofiber substrate that can be used. In addition, certain direct immobilization methods such as epoxide opening mechanism result in almost complete loss of enzymatic activity. The introduction of crosslinkers enables immobilization onto a wide range of nanofiber substrates; frequently used crosslinkers include EDC/NHS, CDI, and glutaraldehyde. The type of crosslinkers used depends on the functional groups on the polymer used, typically a -COOH or -NH_2_ group. Projects that utilized crosslinkers immobilized a wider range of biomolecules including enzyme, antibodies, protein and aptamers. Though there was some activity loss upon immobilization, enzymes were able to retain up to 90% based on the crosslinker used. Currently an extensive study has not been performed to quantify the effect of immobilization on non-enzyme biomolecules, but researchers note uniform distribution of these biomolecules and increased cell capture and proliferation after immobilization. Recently the use of click chemistry has gained attention due to its bioorthogonal chemistry and production of stable covalent bonds; this results in biomolecules retaining their bioactivity. Though more research needs to be done, the current work being done using model biomolecules is promising and is a strong candidate for effective biomolecule immobilization. The use of biomolecules in practical application will continue to grow over the upcoming decades; nanofibers will continue to serve as an excellent substrate for immobilization loading, efficiency and stability.

## Figures and Tables

**Table 1:** Summary of polymers (left column) used in the specified immobilization methods (top row) for covalently attaching biomolecules to nanofiber.

	Direct Immobilization	Direct Immobilization After Surface Modification	EDC/NHS	CDI	GA	Combined	Click
polyacrylonitrile (PAN)		[61,65,66,67,68,69]	[88,94,95,96]		[35,153,154,155,156,160]		
chitosan			[103,104,109]	[116]	[129,130,143,144,145,146]	[136,167]	[196]
polycaprolactone (PCL)			[73,74,102]				[173,176,177,195,202,203]
regenerated cellulose (RC)	[50]	[71,72]	[105]		[149]		[185,187]
poly(acrylic acid) (PAA)			[41]	[3,113,114,117]	[165]		[186]
poly(styrene-co-maleic anhydride) (PSMA)	[39,54,55,56,57,58]					[168]	
nylon					[4,47,135,150,151,152]	[136]	
polyethyleneimine (PEI)			[38,108]		[159,161,162,163]		
poly(glycidyl methacrylate) (PGMA)	[45,46,48]					[169]	[190]
poly(vinyl alcohol) (PVA)				[115,116]	[148,158]		
poly lactic acid (PLA) or poly(L-lactide) (PLLA)			[82]				[178,201]
cellulose acetate (CA)					[164]		[171,175]
poly(methyl methacrylate) PMMA			[34,83]				
poly(m-anthranilic acid) (P3ANA)			[84,85]				
polyhydroxyalkanoate (PHB)			[98,101]				
polyethersulfone (PES)			[99,100]				
silk					[131]		[194]
poly-(6-O-vinylsebacoyl d-glucose) (OVSEG)	[51]						
poly(acrylonitrile-co-2-hydroxyethyl methacrylate) (PAN-c-HEM)	[52]						
polyaniline (PANI)			[34]				
poly(methyl vinyl ether-alt-maleic anhydride) (PMVE/MA)			[87]				
poly(carboxybetaine methacrylate) (pCBMA)			[103]				
poly(lactic-co-glycolic acid)-poly(ethylene glycol)-amine (PLGA-b-PEG-NH_2_)			[106]				
zein					[132,133]		
alginate					[147]		
polyacrylamide (PAAm)					[158]		
feather polypeptide (FP)						[166]	
ethyl cellulose (EC)							[172]
poly[di(propargylamine)phosphazene] (PDPAP)							[174]
4-vinylbenzyl chloride and glycidyl methacrylate (PVBC-b-PGMA)							[179]
poly(2,6-dimethyl-1,4-phenylene oxide)							[180]
poly(vinyl chloride) (PVC)							[181]
poly(ester urea) (PEU)							[182]
furfuryl methacrylate (FuMA)							[183]
poly(γ-benzyl-l-glutamate) (PBLG)							[188]
poly(3-(fluorosulfonyl)propyl methacrylate) (PFPM)							[189]
5-methyl-5-propargyloxycarbonyl-1,3-dioxan-2-one (MPC)							[191]

## Abbreviations

**Acronym**	**Name**
(P(LA–co–MPC))	5-methyl-5-propargyloxycarbonyl-1,3-dioxan-2-one copolymer
AChE	acetylcholinesterase
AEM	N-(2-aminoethyl)maleimide
APTES	3-aminopropyltriethoxysilane
ATRP	atom transfer radical polymerization
BMP	bone morphogenetic proteins
BPA	bisphenol A
BSA	bovine serum albumin
CA	covalent conjugation
CA-125	cancer antigen 125
CD44	cluster of differentiation 44
CDI	1,1′-carbonyldiimidazole
CTC	circulating tumor cell
CuAAC	copper-catalyzed azide alkyne cycloaddition
Cy5	streptavidin
DBU	1,8-diazabicyclo[5.4.0]undec-7ene
DIBO	4-dibenzocyclooctynol
DNA	deoxyribonucleic acid
EDC	1-ethyl-3-(3-dimethylaminopropyl)carbodiimide
EGS	ethylene glycol-bis(sulfosuccinimidyl succinate)
ELISA	enzyme-linked immunosorbent assay
EPC	enzyme aggregation
EpCAM	anti-epithelial cell adhesion molecule
ETBFMS	electrospun triple-blend fibrous mats
FITC	fluorescein isothiocyanate
FMOC	fluorenylmethoxycarbonyl
FP	feathered polypeptide
GA	glutaraldehyde
GRGDS	Gly-Arg-Gly-Asp-Ser
hexDA	hexamethylenediamine
hIgG	immunoglobulin G
HRP	horseradish peroxidase
IgG	Immunoglobulin G
ITO	indium tin oxide
LOD	limit of detection
MA	methylacrylate
MICs	minimum inhibitory concentrations
MNFs	magnetic nanofibers
MWCNTS	multiwall carbon nanotubes
nHA	nanometric hydroxyapatite
NHS	N-Hydroxysuccinimide
NHS-Mal	3-(maleimido) propionic acid N-hydroxysuccinimide ester
NT	nitrocellulose
OVSEG	poly-(6-O-vinylsebacoyl d-glucose)
P3ANA	poly(m-anthranilic acid)
PAA	poly(acrylic acid)
PAAM	polyacrylamide
PAN	polyacrylonitrile
PANI	polyaniline
PANnFs	polyacrylonitrile nanofibers
PBS	phosphate buffered saline
pCBMA	poly(carboxybetaine methacrylate)
PCL	polycaprolactone
PEG	poly(ethylene glycol)
PEGDA	PEG diamine
PEI	polyethyleneimine
PEO	poly(ethylene oxide)
PGMA	poly(glycidyl methacrylate)
PHB	polyhydroxyalkanoate
PIII	plasma-immersion ion implantation
PLA	poly lactic acid
PLGA	poly(lactic-co-glycolic acid)
PLLA	poly(L-lactide)
PMMA	poly(methyl methacrylate)
PMMA CEA	poly(methyl methacrylate-co-ethyl acrylate)
PNIPAM	poly (n-isopropylacrylamide)
PS	polystyrene
PSBMA	poly(sulfobetaine methacrylate)
PSMA	poly(styrene-co-maleic anhydride)
PTFE	Polytetrafluoroethylene
PVA	polyvinyl alcohol
PVP	poly(vinyl pyrrolidone)
QDs	quantum dots
rGO	reduced graphene oxide
rhBMP-2	recombinant human bone morphogenetic protein-2
rhEGF	recombinant human epidermal growth factor
SMCC	succinimidyl 4-(N-maleimidomethyl)cyclohexane-1-carboxylate
SPAAC	strain promoted azide-alkyne cycloaddition
THF	tetrahydrofuran
VD	vitamin-D3
YIGSR	Tyr-Ile-Gly-Ser-Arg
